# From healing to leading: a case study of a community-led mental health program for forcibly displaced people

**DOI:** 10.3389/fpubh.2026.1850816

**Published:** 2026-07-15

**Authors:** Flora Cohen, Kevin Yau, Jane Kennedy, Hayley Saxby, Mozhgan Moarefizadeh

**Affiliations:** 1 School of Social Work, University of Illinois Urbana-Champaign, Champaign, IL, United States; 2 Counseling Department, Asian Theological Seminary, Manila, Philippines; 3 Refugees and Asylum Seekers Information Center (RAIC), Jakarta, Indonesia; 4 Give Freedom International, Newcastle, NSW, Australia

**Keywords:** community-led, Indonesia, mental health, psychosocial support, refugee

## Abstract

There are over 120 million forcibly displaced people in the world, with numbers steadily increasing. The majority of refugees and asylum seekers are displaced in urban settings with fragmented access to health services, and those living in countries without refugee protections face particularly dire circumstances. High rates of trauma exposure and related mental disorders persist alongside structural barriers to care, underscoring an urgent public health equity challenge. This community case study describes the Cope program, a refugee community-led mental health and psychosocial support (MHPSS) initiative implemented by the Refugees and Asylum Seekers Information Center (RAIC) in Jakarta, Indonesia. Using an apprenticeship model, community members transition from program participants to trained, supervised facilitators across three phases (*n* = 30 at baseline, *n* = 16 at endline). Pre- post-assessments using validated measures found significant reductions in depressive symptoms (52%), anxiety symptoms (50%), and Post-traumatic Stress Disorder (PTSD) symptoms (46%) from baseline to endline, with large effect sizes. These findings demonstrate that community-led, culturally grounded MHPSS programming can produce meaningful mental health gains among protractedly displaced populations in non-signatory countries. Beyond individual-level outcomes, the Cope model illustrates how Refugee-Led Organizations (RLOs) can serve as sustainable pillars of inclusive health system delivery—a critical consideration for governments, UNHCR, and global health actors working to advance universal health coverage for displaced populations.

## Introduction

Complex emergencies create significant psychological and social stress on individuals, families, and communities. Refugees and asylum seekers not only experience atrocities in their homes or countries of origin, they typically experience ongoing stressors during and post-flight. Loss of identity and role fulfillment, loss of loved ones, family separation, barriers to economic wellbeing, experiences of stigma and discrimination from host communities, restrictive national policies, and poor living conditions have profound impacts on wellbeing (1–3). These hardships can exacerbate existing mental health disorders, and generate new psychological distress, including an increased prevalence of depression, anxiety, and post-traumatic stress disorder (4).

The global scale of forced displacement has made mental health and psychosocial support (MHPSS) an increasingly urgent humanitarian priority. The Inter-Agency Standing Committee (IASC) Guidelines on Mental Health and Psychosocial Support in Emergency Settings outline a pyramid of care, in which the majority of affected populations can be supported through community- and family-based interventions at the base of the pyramid, with progressively fewer individuals requiring specialized clinical care at higher levels (5). Despite this framework, MHPSS programming in humanitarian settings has historically been concentrated at the clinical level, leaving the foundational layers inadequately resourced. This gap is particularly acute in urban displacement contexts, where refugees live dispersed across cities, often outside of formal camp structures, and are substantially harder to reach through conventional humanitarian programming (6).

Urban refugees face a distinct set of vulnerabilities. Currently, approximately 60% of all refugees and 80% of all internally displaced people live in urban areas (7). While urban refugees may have increased access to the opportunities that cities provide, they also face relative invisibility compared to refugees in camp environments. They lack access to legal protections, must navigate complex urban environments, and face limited resources and rights such as access healthcare, and access schools. Additionally, urban refugees may face stigma and discrimination from their host communities, while simultaneously contending with fragmented social support (8–12). These limitations can cause additional distress for displaced communities, and make both formal and informal mental health program access even more challenging (11).

The majority of refugees (68%) are hosted in low and middle income countries [LMICs (13)]. Several of the world's largest refugee-hosting and refugee-producing states are LMICs who have not ratified the 1951 Refugee Convention or its 1967 Protocol, notable examples include India, Pakistan, Bangladesh, Lebanon, Jordan, Iraq, Libya, Malaysia, Indonesia, and Saudi Arabia (14). This leaves displaced individuals without formal legal status or entitlements. In this non-signatory states, refugees and asylum seekers are often legally barred from working, accessing public education, or receiving social welfare benefits. This systematic dehumanization leads to conditions of chronic material deprivation and exacerbate mental health needs.

Within urban refugee contexts, in LMIC, mental health systems are rarely equipped or accessible. Barriers to care are multilayered, and include financial costs, geographic distance, a shortage of culturally and linguistically competent providers, stigma, and a general absence of trauma-informed services adapted to refugee experiences (15). Even where services normally exist, they frequently fail to reach the communities that need them the most. This treatment gap has accelerated interest in task-shifting and community-based models of care, which redistribute the delivery of evidence-based psychosocial support from specialists to trained lay community members. Task-shifting approaches have demonstrated efficacy across a range of low-resource settings, and evidence is growing for their application in humanitarian and refugee contexts.

One promising mechanism for structuring task-shifting in community-based MHPSS is the apprenticeship model. Rooted in situated learning theory, apprenticeship models develop competency through a graduated process of observation, guided practice, and increasing autonomy within authentic community contexts, rather than through formal didactic instruction (16). In humanitarian MHPSS settings, apprenticeship approaches typically involve an external expert who identifies and mentors promising community members for supervisory roles, who in turn support frontline community facilitators in the delivery of psychosocial programming (17, 18). This cascading design enables programs to build local capacity iteratively, so that each cohort of trained facilitators can go on to support the next, reducing dependence on external expertise over time. This has important implications for sustainability, cultural adaptation, and the long-term institutionalization of MHPSS capacity within displaced communities. While the apprenticeship model has been applied in several global mental health initiatives, its use within Refugee-Led Organizations (RLOs) and urban displacement settings remains underexplored.

Alongside task-shifting, there has been growing recognition of the importance of community participation in the design and delivery of MHPSS programming. Participatory approaches acknowledge that crisis-affected communities possess existing knowledge, strengths, and social networks that are essential to sustainable and contextually appropriate care (19). The World Health Organization and major humanitarian actors have increasingly endorsed community-led models as imperative, particularly in settings where external resources are limited (57). Refugee-Led Organizations have emerged as vital actors in filling service gaps left by underfunded international bodies and national governments, including the retrenchment of international aid. Community members are better positioned to mobilize existing local resources, advocate for contextually appropriate community strengthening activities, and provide information about the best methods for communicating with their peers. There is also considerable evidence that in areas with minimal mental health services (including humanitarian settings) people tend to seek support through extensive, multigenerational family networks. While many people use herbal medications, home remedies, and traditional healers, others use their social capital to access psychoeducation and the minimal services that are available, including support through the humanitarian sector (20). If social networks are not consulted throughout the program design process, the limited resources in humanitarian contexts will not be optimally utilized to meet the demands of the community. As organizations run by and for displaced people, RLOs bring unique contextual knowledge, cultural legitimacy, and community trust that external organizations struggle to replicate (21–23).

However, there is a debate in the field of MHPSS about the involvement of crisis-affected persons in program implementation due to a fear of re-traumatization. While there may be mental health professionals who have experienced displacement and can manage mental health programs, the most common approach to involving crisis-affected populations in implementation is through task shifting. Task shifting or task sharing, which involves extending the scope of interventions to existing cadres of health workers in order to allow for a redistribution of tasks, has been commonly used in health programs (24). Through this approach, tasks are shifted from a highly specialized workforce to less specialized health workers usually from the community itself, typically through manualized protocols. However, the use of task shifting in MHPSS is lesser known. A recent review found that there is a paucity of evidence about the effectiveness of task shifting in MHPSS, its related detriments (such as compounded vicarious trauma and concerns with confidentiality among insular refugee networks), and mechanisms to support cadres engaged in task shifting approaches (25).

It is also important to highlight that task-shifting approaches to implementation, while often seen as the panacea for integrating local participation and expanding the reach of interventions, do not directly influence the program's development, delivery, and evaluation in a participatory manner (25). Often, the people who are trained to deliver interventions are trained in ontologies from the Global North, and requested to shape them with local understandings (26). They also have limited opportunities to provide feedback about the implementation process. Current task-shifting approaches are therefore not the magic answer to the lack of participatory approaches to MHPSS intervention delivery (25).

Overall, evidence on the implementation and outcomes of community-led MHPSS programming in urban refugee settings remains sparse. Much of the existing literature focuses on camp-based interventions, where populations are more geographically concentrated and humanitarian infrastructure is more established. Research in urban displacement contexts is comparatively limited, and there is a particular gap in documentation of how RLOs can sustainably deliver psychosocial support with limited funding, in non-signatory countries, and among populations experiencing protracted displacement with no clear resettlement pathway. The purpose of this paper is to highlight an alternative and sustainable method of implementing MHPSS programming for Refugees and Asylum Seekers experiencing protracted displacement in an urban setting. We will describe the constraints of the setting and the adapted implementation strategy to ensure that key concerns in the refugee and asylum seeking community are addressed. Through these methods, we aim to highlight community strengths and resilience as untapped resources for the provision of high quality programming. We will also explore current barriers to scaling up similar community-led initiatives.

## Methods

### Setting

Currently, Indonesia hosts 12,710 refugees and asylum seekers from over 40 countries (27). The majority of the refugee and asylum seeking population in Indonesia is from Afghanistan (53%), Somalia (10%), Myanmar (9%), Iraq (5%), and Sudan (4%) (27). Indonesia is not a signatory to the 1951 UN Convention or the 1967 Protocol to the Status of Refugees, meaning that the rights of refugees and asylum seekers are not guaranteed. The Indonesian government agrees not to remove individuals registered with United Nations High Commissioner for Refugees (UNHCR), but provides minimal support for refugees and asylum seekers awaiting resettlement to a third country. Given the legal status of refugees in Indonesia, members of the refugee and asylum seeker community do not have access to housing, social security, work rights, or education. Many displaced people living in Indonesia have protested these conditions with UNHCR, however, UNHCR Indonesia is grossly under-funded, receiving just 3% of it's requested budget in 2023 (27, 28). Some Non-Governmental Organizations (NGOs) fill service gaps, however, RLOs provide the largest support to the refugee and asylum seeking community. Refugee-led learning centers cater to refugee needs throughout the Indonesian archipelago. However, these organizations are burdened by unsustainable funding, inadequate resources, and a lack of recognition from the national education system.

Recent evidence shows high rates of exposure to deprivation and potentially traumatic events within the refugee and asylum seeking population in Indonesia. Approximately 72% of community members report experiencing a lack of food or water, 65% experienced a lack of shelter, 59% experienced ill health without access to medical care, and 53% even reported being close to death (29). These findings contribute to a high prevalence of PTSD and depressive symptoms (29). However, an extremely limited number of refugees and asylum seekers have access to irregular mental health counseling with trained professionals. Additionally, where support is available, there are cultural, linguistic, and financial barriers to accessing care. Meanwhile, suicides continue to increase, especially among single male refugees and asylum seekers (30).

### Organization description

Refugee and Asylum seekers Information Center (RAIC) Indonesia is a RLO founded in 2017 in Jakarta. RAIC aims to “break the mold” through providing support that is directly developed from community-described needs. Through the provision of basic needs, healthcare, mental health services, and legal information, RAIC aims to support refugee and asylum seeking community members holistically and sustainably. RAIC believes that all programming should respond to real community needs. The majority of RAIC staff are refugees themselves, and the staff is mostly women. Volunteers and staff, as members of the refugee and asylum seeker communities themselves, understand the struggles, needs, and barriers faced by refugee and asylum seeker communities in transit through their own experiences.

### Intervention description

Refugee and Asylum seekers Information Center developed the Cope program (“Cope”) in 2018 to address refugee community mental health needs in Indonesia. Cope was originally developed to meet the needs of refugees living in urban settings, rural settings, and in detention centers. The program was developed in collaboration with the refugee community living in Indonesia and experts in refugee mental health. External experts, who had worked extensively with the local community, devised a program that addressed common mental health issues experienced by the refugee communities. This program was pioneered among a small group of participants recruited through social media and referrals. With the input of the refugee participants, the program was further contextualized to include coping skills, mental health and suicide first aid, and communication skills. It was also adapted to include a decreased group size to five participants, a change in the length of the program to ten sessions, and a co-facilitation model was utilized. This program was then manualized and translated, with the help of the original participants, into various languages used by the community.

The Cope Program employs an apprenticeship model, which allows for continuous and on the job training. The apprenticeship model employs the use of external trainers, supervisors from the local community, and community members as counselors. Within the apprenticeship model, a trainer from outside of the local context helps to identify local community members who would suit a more advanced role (supervisor) through training (18). External experts mentor and coach those identified as superiors for local community members identified as facilitators. The program is iteratively developed based on changing community needs and perceptions collected through iterative community verbal and written feedback.

**Figure 1:**
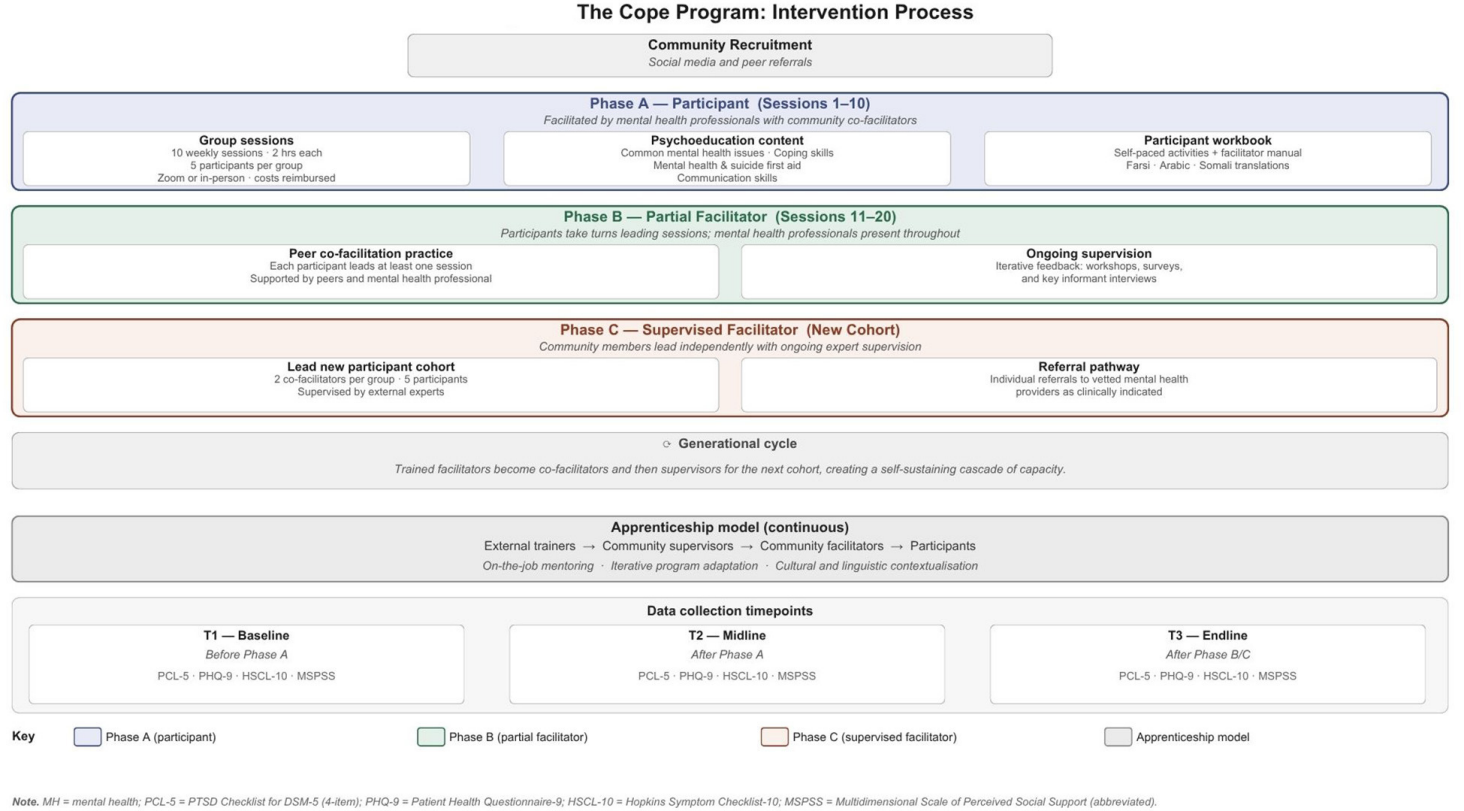
The cope program: intervention process.

Through the apprenticeship model, community members are invited to attend a series of peer-led psychoeducation workshops and activities discussing common mental health issues experienced by refugee communities, coping skills, mental health and suicide first aid, and communication skills (Figure 1). Cope includes 10 sessions of psychoeducation and activities to address the multifaceted needs of refugees in the Indonesian context. Every participant is provided with a self-paced workbook with activities, and a manual in order to participate in and facilitate Cope sessions. Each cohort has co-facilitators (2 per group) who lead 10 sessions (each session lasts 2 h long, they are held once per week, and there are five participants per group). After the 10 sessions, participants are invited to learn and practice facilitation skills with their group members for 10 more sessions. Then, they are invited to become co-facilitators themselves with a new cohort of participants. The cycle continues with a generational design, where local community members are trained, mentored, and supported to become local experts in the program.

The aim of Cope is to overcome barriers refugees and asylum seekers face in accessing quality mental health services, through the provision of free, accessible, and sustainable psychosocial support. Cope utilizes low-cost multimedia technology to expand access to services, wherein participants can either join via zoom or in-person within their own communities. Furthermore, the program is free to all participants, and internet or travel expenses are reimbursed. Through iterative development, the program is designed to address concerns specific to the refugee and asylum seeking community in Indonesia. Furthermore, the workbook and manual has been translated into the main languages of the community, Farsi, Arabic, and Somali in order to accommodate participant linguistic concerns.

In the first phase of the program (A) participants join groups that are facilitated by mental health professionals with extensive experience in group facilitation. In the next phase (B) the participants take turns leading group sessions, with the support of mental health professionals during the sessions. Then in the final phase (C) facilitators lead their own groups with a new cohort of participants, and ongoing supervision with mental health professionals. Through participation in phase A of the program as participants, phase B as partial facilitators (taking turns as facilitators with their peers), and phase C as supervised facilitators, community members are trained to implement psychosocial support in their own communities. This support is therefore sustainable, culturally adapted, and supported by trained supervisors. The program empowers community members to be advocates for community mental health, following the first and second layers of the MHPSS pyramid. Where participants require individualized support, referrals are also made available to vetted mental health providers as needed. An emphasis on obtainable, affordable care is a fundamental strength of Cope, as its primary goal of attainable psychosocial support is fully realized through its focus on accessibility.

## Data

### Ethics statement

This study involved the secondary analysis of data collected as part of a routine program evaluation and did not constitute human subjects research requiring Institutional Review Board (IRB) review under applicable regulatory guidelines. As this evaluation was conducted for internal quality improvement and program monitoring purposes, prior ethics committee approval was not required. Nevertheless, all participants were fully informed about the nature of the data collection and its potential use for research purposes. Written and verbal informed consent was obtained from all participants prior to their enrollment. Participation was entirely voluntary, and participants were free to withdraw at any time without consequence. All data were handled in accordance with principles of confidentiality and participant protection consistent with the Declaration of Helsinki.

### Data collection

Data was collected at three time points, before enrolling in a Cope group (t1), following completion of the first phase of the program (t2), and following completion of the second phase of the program (t3). Participants were interviewed by three trained research assistants who included an undergraduate student, administrative staff, and a mental health provider, with the support of community interpreters as needed. Each research assistant completed a training on research ethics, to ensure the safety and integrity of the study. Interpreters supported participants who spoke either Somali, Arabic, or Farsi. All interviews were conducted over zoom. The interviewer asked the questions, and answered them in a Microsoft form. If the participant was interested in viewing the survey themselves, the screen was shared during the session. However, the majority of participants did not view the survey.

### Variables

The Post-traumatic Stress Disorder Checklist (PCL-5) was used to measure symptoms of Post-traumatic Stress Disorder (PTSD). The PCL-5 is a self-report questionnaire designed to assess the symptoms of PTSD over the past month, as outlined in the DSM-5. An abbreviated four item version of the 20-item PCL-5 was used, which has been used as a valid and reliable tool for measuring PTSD symptoms in highly-traumatized populations (31, 32). Each item is rated on a 4-point scale from 0 (Not at all) to 4 (Extremely). The total score ranges from 0 to 16, with higher scores indicating more severe PTSD symptoms. The Cronbach's alpha (*a*) = 0.78, indicating a reliable and consistent measure of PTSD symptoms. The PCL-5 has been validated with similar refugee populations (33).

The Patient Health Questionnaire (PHQ-9) was used to assess the presence and severity of depressive symptoms over the past 2 weeks (34, 35). It aligns with the DSM-5 criteria for diagnosing major depressive disorder. Each of the nine items is scored from 0 (Not at all) to 3 (Nearly every day), resulting in a total score ranging from 0 to 27. Higher scores indicate greater depression severity. Specific score ranges suggest different levels of depression, guiding potential treatment recommendations. The PHQ-9 is a valid and reliable instrument for evaluating depressive symptoms among forcibly displaced populations (36–38). The Cronbach's alpha (*a*) = 0.79, indicating a reliable and consistent measure of depressive symptoms.

In order to evaluate the presence of psychological distress over the past week, we used the Hopkins Symptom Checklist (HSCL-10). The HSCL consists of four items related to anxiety and six related to depression, which collectively indicate psychological distress. All items have four response categories, ranging from 1 (Not at all) to 4 (Extremely). Response values are summed, and divided by the number of items (range 1–4), higher scores indicate a higher symptom load. The HSCL-10 has previously shown validity and reliability as a measure of distress among forcibly displaced populations (39–41). The Cronbach's alpha (*a*) = 0.88, indicating an excellent reliable and consistent measure of psychological distress symptoms.

The abbreviated Multidimensional Scale of Perceived Social Support Scale (MSPSS) was used to assess participant perceptions of social support from significant others, family, and friends. The abbreviated MSPSS has three items which are rated on a 7-point Likert scale. Statements are read, and participants respond whether they agree or disagree with 1 indicating “Very Strongly Disagree” and 7 indicating “very strongly agree.” Higher scores represent greater perceived social support (42). The MSPSS is a reliable and valid measure of social support with forcibly displaced populations (43, 44). The Cronbach's alpha (*a*) = 0.50, indicating a poor measure of social support, however, this test may not be a reliable indicator due to the small number of questions included in this tool.

### Statistical analysis

First, we assessed demographic characteristics, including age, gender, country of origin, and the use of formal mental health services. Then, we used paired-sample *t*-tests to evaluate changes in outcomes across time. This approach was selected because participants completed assessments at repeated time points. We were specifically interested in within-person differences rather than between-group comparisons. For each outcome variable, paired *t*-tests compared scores at baseline with subsequent time points (midline, and endline). Normality of the difference scores was assessed using Shapiro-Wilk tests and inspection of Q-Q plots. In cases where the normality assumption was violated, we conducted sensitivity analyses using the non-parametric Wilcoxon signed-rank test.

Effect sizes were calculated using Cohen's d for paired samples (d = mean difference/standard deviation of differences) to quantify the magnitude of change. To account for multiple comparisons across time points, we applied a Bonferroni correction, adjusting the threshold for statistical significance accordingly. All analyses were conducted in Stata 18. Results are reported as mean differences with 95% confidence intervals (CIs) and corresponding *p*-values. According to de Winter (45) using a paired *T*-test should not be contested in a small sample, if there is a high within-pair correlation. Using paired *t*-tests is a reliable strategy in clinical settings, where considerably large samples are sometimes broken down by variables such as age and gender, making the sample considerably smaller.

## Results

The majority of participants in the original sample (*n* = 30) were from Afghanistan (66.7%; *n* = 20), Somalia (20%; *n* = 6), Iran (1%; *n* = 3), and Pakistan (0.03%; *n* = 1). The final sample followed similar characteristics, with 13 participants from Afghanistan (81.25%), 2 from Iran (12.5%), and one from Somalia (6.25%). There was significant attrition in the sample due refugee relocation and scheduling conflicts. Facilitators were encouraged to support retention through collaborating to find the best times to meet, however, there were limitations.

Participants were predominantly women, with 24 women at baseline, 25 women at midline, and 20 women at endline (Table 1). There were 6 men at baseline, 5 men at midline, and 3 men at endline. The majority of participants were below 27 years old, throughout baseline, midline, and endline. However, due to significant attrition after the first phase of the program, additional participants were added in the second phase, contributing to a different distribution in demographic characteristics. Additionally, most participants were not formally employed, but they were volunteering at children's learning centers in the community. In the baseline sample 25% had experienced some formal mental health services, however, at endline only 4.5% had received formal individual support.

**Table 1:** Demographic characteristics of participants at each timepoint.

Characteristic	Baseline, *n* = 30	Midline, *n* = 30	Endline, *n* = 23
Country of origin			
Afghanistan	63.3% (*n* = 19)	66.7% (*n* = 20)	82.6% (*n* = 19)
Somalia	20.0% (*n* = 6)	20.0% (*n* = 6)	8.7% (*n* = 2)
Iran	10.0% (*n* = 3)	6.7% (*n* = 2)	8.7% (*n* = 2)
Pakistan	3.3% (*n* = 1)	–	–
Afghanistan/Iran	3.3% (*n* = 1)	6.7% (*n* = 2)	–
Gender			
Women	80.0% (*n* = 24)	83.3% (*n* = 25)	87.0% (*n* = 20)
Men	20.0% (*n* = 6)	16.7% (*n* = 5)	13.0% (*n* = 3)
Age group			
18–27	73.3% (*n* = 22)	63.3% (*n* = 19)	65.2% (*n* = 15)
28–37	10.0% (*n* = 3)	23.3% (*n* = 7)	13.0% (*n* = 3)
48–57	16.7% (*n* = 5)	13.3% (*n* = 4)	21.7% (*n* = 5)
Occupation			
Volunteer teacher/learning center	33.3% (*n* = 10)	45.0% (*n* = 14)	56.5% (*n* = 13)
Student	6.7% (*n* = 2)	13.3% (*n* = 4)	8.7% (*n* = 2)
Interpreter/translator	6.7% (*n* = 2)	8.3% (*n* = 2)	8.7% (*n* = 2)
Other/not employed	53.3% (*n* = 16)	33.3% (*n* = 10)	26.1% (*n* = 6)
Receiving formal mental health services			
Yes	20.0% (*n* = 6)	6.7% (*n* = 2)	4.3% (*n* = 1)
No	80.0% (*n* = 24)	93.3% (*n* = 28)	95.7% (*n* = 22)

Demographic distributions are based on all participants present at each timepoint. Attrition occurred primarily due to refugee relocation and scheduling conflicts. Country spellings standardized from open-text responses.

Notably, the first phase of the program was participation in the groups while a mental health professional facilitated the sessions (Figure 1 and Table 2). Participants experienced significant decreases in mental distress following the first phase of the program. The most dramatic decrease was between baseline and midline, where the mean of depressive symptoms dropped by 40% among participants. Symptoms of PTSD were similarly significantly reduced, with a 45% reduction in symptoms among participants between baseline and midline. Symptoms of anxiety were significantly reduced at a rate of 40% between baseline and midline. Lastly, perceived social support among participants did not significantly change, although there were improvements in social support.

**Table 2:** Paired sample *t*-test results for outcome measures across timepoints.

Scale	Baseline		T1→T2: baseline to midline						T1→T3: baseline to endline				
	M (SD)	*n*	M (SD)	MD	95% CI	d	*p*	*n*	M (SD)	MD	95% CI	d	*p*
PCL-5	8.55 (5.14)	22	4.82 (4.46)	3.73	[1.73, 5.72]	0.78	0.002^**^	18	5.78 (4.67)	2.67	[0.56, 4.77]	0.59	0.027
PHQ-9	11.95 (5.48)	21	6.81 (5.34)	5.14	[2.76, 7.52]	0.92	< 0.001^***^	17	6.18 (5.09)	6.06	[3.11, 9.00]	0.98	0.001^**^
HSCL-10	13.14 (4.87)	22	7.50 (4.98)	5.64	[2.99, 8.28]	0.89	< 0.001^***^	18	6.22 (4.82)	6.28	[4.21, 8.35]	1.40	< 0.001^***^
MSPSS	15.18 (5.03)	22	16.73 (4.72)	−1.55	[−3.38, 0.29]	−0.35	0.122	18	17.11 (4.79)	−1.00	[−3.26, 1.26]	−0.20	0.411

MD, mean difference (baseline minus follow-up; negative values for MSPSS indicate improvement); 95% CI, 95% confidence interval; d, Cohen's d for paired samples; PCL-5, PTSD Checklist for DSM-5 (4-item); PHQ-9, Patient Health Questionnaire-9; HSCL-10, Hopkins Symptom Checklist-10; MSPSS, Multidimensional Scale of Perceived Social Support (abbreviated, 3 items). Bonferroni-corrected significance threshold *p* < 0.025 for two comparisons per outcome.

^*^p < 0.025. ^**^p < 0.010. ^***^p < 0.001.

Participants also experienced significant decreases in mental health symptoms from midline to endline (Phase B to Phase C), where they were trained to facilitate sessions through intensive training, supervision, and support while they co-lead groups. From baseline to endline (Phase A to Phase C) there were significant reductions in depressive symptoms (52%), anxiety symptoms (50%), and PTSD symptoms (46% decrease). There were increases in social support, however, it did not reach a level of significance.

Subgroup analyses were conducted by gender and age group, however, these findings should be interpreted with caution given the small and unequal subgroup sizes. The 28–37 age group (*n* = 5) had insufficient matched pairs to support statistical testing and is therefore excluded from subgroup reporting. Among women (*n* = 17–17 paired at T1–T2; *n* = 14–15 at T1–T3), significant improvements were observed across all three symptom measures. Depressive symptoms (PHQ-9) decreased significantly from baseline to midline (M = 11.88 to M = 5.88; *d* = 1.05, *p* = 0.001) and from baseline to endline (M = 12.29 to M = 6.71; *d* = 0.85, *p* = 0.009). PTSD symptoms (PCL-5) similarly decreased from baseline to midline (M = 8.53 to M = 4.18; *d* = 0.98, *p* = 0.001) and baseline to endline (M = 9.13 to M = 6.20; *d* = 0.73, *p* = 0.016). Psychological distress (HSCL-10) decreased significantly at both timepoints (T1–T2: *d* = 0.76, *p* = 0.007; T1–T3: *d* = 1.44, *p* < 0.001). Social support (MSPSS) showed small improvements that did not reach statistical significance at either timepoint (*p* = 0.180; *p* = 0.374). Among men (*n* = 5–6 paired), the male subsample was too small to support reliable inference. A significant decrease in psychological distress was observed from baseline to midline (*d* = 1.73, *p* = 0.026), and a large effect size for depressive symptoms was observed from baseline to endline (*d* = 2.53), though this did not reach conventional significance (*p* = 0.070) with only three matched pairs.

Among participants aged 18–27 (*n* = 15–16 paired), significant reductions were observed in PTSD symptoms (T1–T2: *d* = 0.75, *p* = 0.011), depressive symptoms (T1–T2: *d* = 0.78, *p* = 0.011), and psychological distress (T1–T2: *d* = 0.83, *p* = 0.006; T1–T3: *d* = 1.08, *p* = 0.004). Reductions in PTSD and depressive symptoms from baseline to endline showed medium-to-large effect sizes but did not reach significance after attrition (*p* = 0.350 and *p* = 0.068 respectively). Among participants aged 48–57 (*n* = 4–5 paired), large effect sizes were observed across outcomes, with significant reductions in depressive symptoms from baseline to endline (M = 16.80 to M = 6.00; *d* = 2.38, *p* = 0.009) and psychological distress (T1–T3: *d* = 3.44, *p* = 0.002). PTSD reductions at both timepoints showed large effects (*d* = 1.71 and *d* = 1.31) that approached but did not reach significance (*p* = 0.059), likely reflecting limited power with *n* = 4–5. Social support did not change significantly in either age group.

## Discussion

While the wider humanitarian community has stressed the importance of community-led approaches to interventions, in practice, there is limited evidence about community-led programming in MHPSS (46). Meanwhile, crisis-affected individuals may be the most well-positioned to understand their context, culture, history, cultural idioms of distress, and pathways to healing. Community members are motivated and passionate about participating in program development, but are often constrained by the lack of resources provided directly to them and an insufficient literacy in the methods to obtain them (46).

Women in refugee and asylum seeking contexts face compounded vulnerabilities, including gender-based violence, heightened caregiving responsibilities, and social isolation, all of which are known to elevate mental health risk and to complicate help-seeking (47, 48). The Cope program's peer-led, community-embedded design may be particularly well-suited to reaching women, for whom cultural and linguistic barriers to formal mental health services are often most pronounced. The predominantly female staff and volunteer base at RAIC likely further reduced barriers to engagement and disclosure.

Exclusion from participation or leadership, communication barriers, inadequate technology access, and urban growth are factors that have been shown to significantly impact the effectiveness of similar support models. External factors including a refugee's ability to maintain stable housing or finding clean water, present a challenge to both the Cope initiative and humanitarian intervention practice altogether. Exclusionary resettlement policies push this community to the margins, where they are often overlooked and disenfranchised. The project's flexibility in its peer-led focus allows for the project to reach hard to find communities. In Cope's program implementation, participants are supported by members of their own community, using evidence-based psychosocial support practices with multimedia elements.

Previous studies have shown efficacy of guided self-help and task-shifting models of mental health interventions among refugees in camp settings, but research in urban areas is limited, perhaps due to the difficulty of accessing urban refugee communities (25). The Cope Program helps to fill this knowledge gap, working to better understand the psychosocial experience of refugees in Indonesia, and helping develop best-practice strategies for this often “illusive” population. This program seeks to extend current evidence through its unprecedented implementation strategy.

Community-led and participatory research must be handled delicately because it can have potential detriments. If external organizations and researchers only involve existing community leaders in program design and implementation, they can accidentally reinforce damaging hierarchies that disadvantage vulnerable and already marginalized groups (49). Additionally, demographic groups such as those classified by gender, caste, disability, race, HIV status, religious group, affiliations, tribe, clan, and sexual orientation require particular attention in order to ensure the inclusion of relevant actors (50). So, innovative and more inclusive methodologies that extend to traditionally marginalized groups should be utilized. For example, in Lebanon, a group of youth leaders met separately from an overarching community group in order to freely discuss their opinions and appoint representatives to bring them to the larger community action group (51). In northern Kenya, humanitarian actors set up boxes where anyone could write written complaints and send them to the (52)agency. The Cope program utilizes multiple methods of feedback between sessions, including workshops, anonymous online surveys, and key informant interviews.

Genuine relationships that allow for honest participation and collaboration take time to build. They require on-the-ground experience, often before the disaster even begins. However, the current state of humanitarian aid, especially MHPSS, often involves outside researchers parachuting into humanitarian settings for brief periods of time—the antithesis of long term honest participation and collaboration (53). Alternatively, remote-led interventions, which have increased during the COVID-19 pandemic, have diminished collaborative processes (53). Furthermore, even when genuine rapport has been built between researcher or implementer and beneficiary, this does not mean active participation. If researchers or program implementers are not able to engage in critical reflexivity about their roles and identity in collaboration, beneficiaries may not even be comfortable expressing their concerns.

Even with the encouragement of open and inclusive participation from community members, little is known about the financial compensation for participation. If community members are engaged in research and program implementation, financial compensation comparable to their humanitarian worker counterparts is vital. This exploitation of community knowledge and resources, can be additionally burdensome to crisis-affected persons who are already contending with various challenges. For example, VHTs who support care delivery in the Kiryandongo refugee settlement are paid a total of $8 per month, while they are expected to actively manage medical cases in their communities, including follow up with community members, calling ambulances, accompanying patients, and coordinating with organizations. Other task shifting interventions have similarly ignored the financial implications of active participation (25).

There is a remaining question regarding if communities have all the best answers for the development and delivery of programs. For example, in Sri Lanka during livelihood recovery programs, communities chose a particular superior breed of goat to restock their assets. The agency procured these goats and transported them to the programmatic regions. However, the goats were not suited to the terrain and died much quicker than the local breeds (54). Furthermore, regarding mental health services, traditional healing mechanisms for mental illnesses such as *pasung* or chaining in Indonesia, may be the proposed strategies from traditional healers but do not ensure the protection of people with serious mental illnesses according to international mandates (55). In order to combat these concerns, it is vital to integrate the perspectives of mental health professionals alongside community members, in order to ensure interventions are both applicable and adaptable to context.

Lastly, while mental health interventions can theoretically be platforms for participants to voice very real problems in their social ecologies such as discrimination and daily violence, the prescribed nature of evidence-based practices can readily become a tactic for silencing communities (56). To generate more effectiveness, policy makers at national, regional, and international levels should look beyond transplanting external models to the Global South, but rather adopt long-term processes to system design that integrate local idioms of distress, are culturally appropriate, and feasible within the boundaries of available and planned resources.

## Limitations

Several limitations of this study should be considered when interpreting findings. First, the sample size was small (*n* = 30 at baseline, *n* = 16 at endline), and significant attrition occurred due to refugee relocation and scheduling challenges—factors inherent to protracted displacement contexts but which limit statistical power and generalizability. Attrition driven by refugee relocation and scheduling instability is common, and reflects the structural precarity that defines protracted displacement. Future research should explore adaptive delivery models that allow participants to re-enter the program after interruption, pause and resume cohorts flexibly, or receive bridging support between phases, rather than treating dropout as a terminal endpoint. Retention strategies should also be developed collaboratively with community members and tested prospectively, including the use of peer outreach, flexible session scheduling determined by participants themselves, and mobile or asynchronous delivery options for those who relocate.

The markedly lower representation of men in the program also warrants attention. Research consistently identifies single men as among the most at-risk and least-reached subgroups within refugee populations, and the rising rate of suicide among single male refugees and asylum seekers in Indonesia underscores the urgency of developing targeted engagement strategies for this group (29). The challenge of reaching men, and of engaging the most marginalized within already marginalized communities similarly demands deliberate methodological attention in future work. Researchers should consider whether the current group format and psychoeducation approach require adaptation for these subpopulations, or whether complementary program streams are needed.

The absence of a control group in the current study means that causal attributions cannot be made with confidence. External factors, including natural variation over time, concurrent life events, or other community supports, may have contributed to outcomes. Future research should prioritize comparative designs, including waitlist controls, stepped-wedge trials, or matched comparison groups that are feasible within humanitarian settings and respectful of the ethical obligation not to withhold support from those in need. The program phases involved professional mental health facilitation in Phase A and substantial co-facilitation in Phases B and C; the relative contributions of professional involvement vs. community facilitation to observed outcomes cannot be disentangled. Future research using randomized or comparative designs would help isolate program-specific effects. Longitudinal follow-up beyond program completion would also clarify whether the symptom gains observed here are sustained over time, a critical question for any program seeking to demonstrate durable community impact.

Furthermore, outcome data relied on self-report measures administered via interview, which may be subject to social desirability bias, particularly given that interviewers were affiliated with the implementing organization. The Cronbach's alpha for the abbreviated MSPSS (α = 0.50) was also below acceptable thresholds for reliability, and social support findings should therefore be treated as exploratory only. Finally, participants were recruited through social media and referrals, which may have produced a self-selected sample not representative of the broader refugee and asylum seeking community in Indonesia.

## Policy implications

The Cope program offers several lessons for policymakers and humanitarian health actors seeking to advance equitable and inclusive MHPSS within national and global health systems. First, RLOs such as RAIC represent an underutilized but highly effective entry point for sustainable MHPSS delivery. As global humanitarian budgets face increasing pressure, formal recognition and direct, multi-year funding of RLOs can stretch resources further while producing culturally grounded, community-owned outcomes. UNHCR, host governments, and bilateral donors should explore dedicated funding mechanisms for RLO-led MHPSS programming. Second, the apprenticeship model described here offers a replicable framework for expanding MHPSS reach in non-signatory countries where formal health systems provide minimal protections for displaced populations. National ministries of health in countries hosting significant refugee populations could incorporate community-based MHPSS delivery into their universal health coverage strategies, adapting the apprenticeship approach to local contexts and integrating it with existing community health worker systems. Third, the Cope experience underscores that refugees are not merely recipients of care. Refugees are skilled, motivated health actors who can contribute substantially to the communities around them. Policies that enable refugees to work formally in the health sector, including as community health workers, would convert an untapped resource into a systemic asset. Ultimately, sustainable progress on refugee mental health requires moving beyond project-based interventions toward long-term investment in community-owned systems that align with universal health coverage principles. The financial and institutional sustainability of RLO-led programming remains an underexamined area. Research that documents the cost-effectiveness of community-led MHPSS delivery relative to externally-led alternatives, and that identifies which funding mechanisms and institutional partnerships best support RLO capacity over time, would substantially strengthen the evidence base for scaling models like Cope.

## Conclusion

There is global recognition about the need for MHPSS programming, and current programs have made considerable impacts on individual and community wellbeing. However, there is room for growth in the area of community participation for program delivery. It is important to consider the process of implementation from assessment and development to dissemination, and the various methods for community member engagement. Through the utilization of community perspectives, it is possible to continue to improve MHPSS programming.

Community participation should be integrated throughout humanitarian research and programming, including the diagnosis, planning, implementation, monitoring, evaluation, and sustainment phases. In fact, the dismissal of community perspectives can have volatile repercussions. In MHPSS specifically, effectively ignoring community perspectives can lead to further dehumanization and a replication of the lack of agency experienced during forcible displacement. Alternatively, community-led programs can reinforce community efficacy, a vital component to community recovery and growth following crises. Meanwhile, academic institutions and larger humanitarian actors can provide support leveraging access to funding, supporting the use of rigorous research methodologies, and analyzing data in order to generate more sustainable programming collaboratively.

## Data Availability

The original contributions presented in the study are included in the article/supplementary material, further inquiries can be directed to the corresponding author.
